# The Anti-*Campylobacter* Activity and Mechanisms of Pinocembrin Action

**DOI:** 10.3390/microorganisms7120675

**Published:** 2019-12-10

**Authors:** Anja Klančnik, Katarina Šimunović, Jasna Kovac, Orhan Sahin, Zuowei Wu, Darinka Vučković, Maja Abram, Qijing Zhang, Sonja Smole Možina

**Affiliations:** 1 Department of Food Science and Technology, Biotechnical Faculty, University of Ljubljana, Jamnikarjeva 101, SI-1000 Ljubljana, Slovenia; Katarina.simunovic@bf.uni-lj.si (K.Š.); jasna.kovac@psu.edu (J.K.); Sonja.smole@bf.uni-lj.si (S.S.M.); 2 Department of Food Science, The Pennsylvania State University, University Park, PA 16802, USA; 3 Department of Veterinary Microbiology and Preventive Medicine, College of Veterinary Medicine, Iowa State University, Ames, IA 50011, USA; osahin@iastate.edu (O.S.); wuzw@iastate.edu (Z.W.); zhang123@iastate.edu (Q.Z.); 4 Department of Microbiology, Faculty of Medicine, University of Rijeka, Braće Branchetta 20, 51000 Rijeka, Croatia; darinka.vuckovic@medri.uniri.hr (D.V.); maja.abram@medri.uniri.hr (M.A.)

**Keywords:** *Campylobacter jejuni*, pinocembrin, mechanism of antibacterial activity, gene expression, virulence

## Abstract

We investigated the anti-*Campylobacter* activity of pinocembrin and its mechanism of action, as well as *Campylobacter* responses to pinocembrin treatment at the genetic and phenotypic levels, using *C. jejuni* NCTC 11168 and a multidrug efflux system repressor mutant (11168*ΔcmeR*). At its minimal inhibitory concentration, pinocembrin significantly increased cell membrane permeability of *Campylobacter*. Interestingly, at sub-inhibitory concentrations, pinocembrin did not significantly alter membrane functionality and it increased bacterial fitness. Treatment with pinocembrin evoked decreased expression of ribosomal proteins and down-regulation of several NADH dehydrogenase I chain subunits and proteins involved in iron uptake. This suggests altered protein production and redox cycle and iron metabolism. Interestingly, the chelation of Fe ions during the treatment with pinocembrin increased *C. jejuni* survival, although there was no increase in the formation of reactive oxygen species. Pre-treatment of *C. jejuni* with sub-inhibitory concentrations of pinocembrin for 2 h resulted in a 1 log decrease in *C. jejuni* colony forming units in mice liver at 8 days post-infection, compared to untreated *C. jejuni*. These findings suggest that pinocembrin modulates the metabolic activity of *C. jejuni* and that pre-treatment of *C. jejuni* with pinocembrin influences its virulence potential in mice. This anti-*Campylobacter* potential of pinocembrin warrants further investigation.

## 1. Introduction

Campylobacteriosis was the most frequently reported zoonotic disease in humans in Europe over last decade [1]. Diarrhoeal disease and chronic complications of *Campylobacter* infection are common, including reactive arthritis and Guillain-Barré syndrome [2].

The European Centre for Disease Control has reported increased frequencies of antibiotic-resistant pathogenic bacteria and it is actively encouraging alternative strategies to control *Campylobacter* contamination and to combat infections without further increases in bacterial resistance [3]. Reduction of *Campylobacter jejuni* prevalence would decrease the risk of human exposure, which would have substantial positive impact on food safety and public health. Alternative approaches for prevention of *Campylobacter* contamination have been focused on the use of antimicrobials of natural origins. One such product is the 5,7-dihydroxyflavanone, pinocembrin, which is a major flavonoid that is used as a multifunctional active compound in the pharmaceutical industry [4,5,6]. Pinocembrin is found in a wide range of different plant species and is considered to be part of a normal healthy diet. It is commonly found in honey, propolis, and ginger roots [7]. Pinocembrin has a number of known pharmacological activities, including antibacterial activity [5,6]. However, its potential to inhibit *Campylobacter* has not been tested to date.

Resistance of *C. jejuni* to antimicrobials, including phenolic compounds and plant extracts, involves the activity of the main resistance-nodulation-cell-division (RND)-type efflux pump, known as CmeABC [8,9]. This efflux system is regulated through the transcriptional repressor CmeR, which is encoded by a gene that is positioned immediately upstream of *cmeA* and interacts directly with the promoter region of *cmeABC*, to modulate its expression [10]. Recently, Yang et al. [11] and Zhang et al. [12] defined the impact of substitutions, insertions, and deletions in the *cmeR–cmeA* intergenic region of *C. jejuni* and reported that strains with specific polymorphisms are more likely to show increased resistance to tetracycline, doxycycline, florfenicol, chloramphenicol and gentamicin [12,13]. This is due to decreased binding of CmeR to CmeABC, which results in overexpression of the efflux pump.

The aim of this study was to investigate the mechanisms of pinocembrin action in resistant *Camplyobacter* using a *C. jejuni* NCTC 11168 construct with an inactivated efflux system repressor (11168*ΔcmeR*) as a model strain with induced antimicrobial resistance. The effects of pinocembrin on growth kinetics, membrane integrity, oxidative stress induction, and global gene transcription were investigated in vitro. Finally, the potential virulence-modifying effects of *C. jejuni* pre-treatment with sub-inhibitory concentrations of pinocembrin were investigated in vivo through quantification of the bacterial burden in the liver of infected BALB/c mice.

## 2. Materials and Methods

### 2.1. Bacterial Strains and Growth Conditions

The minimal inhibitory concentrations (MICs) were determined for the reference strain *C. jejuni* NCTC 11168 (National Collection of Type Cultures) and for *C. jejuni* NCTC 11168 Δ*cmeR*, which has a disrupted open reading frame of the gene encoding for the efflux repressor *cmeR*, and thus provides a model strain of intrinsic resistance [10]. Additionally, two *C. jejuni* NCTC 11168 mutants with disrupted *cmeB* and *cmeF* genes were also included in the study [9]. Cultures were stored at −80 °C, and grown on Mueller-Hinton agar (Oxoid, Hampshire, UK) with incubations at 42 °C under microaerobic conditions (5% O_2_, 10% CO_2_, in 85% N_2_) for 24 h. The second passage of each culture in exponential growth phase was used in the experiments.

### 2.2. Antimicrobial Susceptibility Testing and Resistance Mechanism

The microdilution method was used to measure the MICs, with optical density measurements at 600 nm (OD_600_) and addition of a bacterial viability indicator (BacTiter-Glo reagent; Promega Corporation, Madison, WI, USA) [14]. The MIC was the lowest concentration where no metabolic activity was observed after 24 h, on the basis of absence of a bioluminescence signal, measured using a microplate reader (Tecan, Mannedorf/Zurich, Switzerland) [14]. Stock solutions of pinocembrin were prepared in dimethyl sulphoxide (DMSO; Sigma-Aldrich, St. Louis, MO, USA). The antimicrobial activity of pinocembrin was tested against *C. jejuni* NCTC 11168 and its efflux pump knockout mutant strains that lack functional genes for the efflux pumps CmeABC (Δ*cmeB*) and CmeDEF (Δ*cmeF*) and for the efflux pump repressor CmeR (Δ*cmeR*). Additionally, the antimicrobial activity of pinocembrin was tested using disc diffusion and agar-well diffusion according to EUCAST 2019 [15]. The contents of the compounds per disc (7 mm diameter) and well (4 mm diameter) were as follows: ciprofloxacin 5 µg, erythromycin 15 µg, and pinocembrin of 100 µg, 50 µg, 25 µg, and 12.5 µg. Ciprofloxacin and erythromycin (Sigma-Aldrich, St. Louis, MO, USA) were used for comparisons of the antimicrobial effects. All of the MIC measurements were carried out in triplicate with appropriate controls.

### 2.3. Time-Kill Kinetics

The time-kill kinetics were determined for *C. jejuni* NCTC 11168 Δ*cmeR* using the broth microdilution method, with incubation at 42 °C under microaerobic conditions, as described previously [16]. Sequential plating of serial culture dilutions was carried out on Mueller-Hinton agar, at 0 h, 3 h, 6 h, and 24 h after addition of pinocembrin at 16 µg/mL (0.25 × MIC), 32 µg/mL (0.5 × MIC), 64 µg/mL (MIC), and 128 µg/mL (2 × MIC). The colony forming units (CFUs) were counted after 24 h incubation at 42 °C under microaerobic conditions [16].

### 2.4. Membrane Integrity

The influence of pinocembrin on the membrane integrity of *C. jejuni* NCTC 11168 Δ*cmeR* was investigated at 16 µg/mL (0.25 × MIC) to 128 µg/mL (2 × MIC), using Live/Dead BacLight Bacterial Viability kits (L-7012; Molecular Probes, Eugene, OR, USA), as reported previously [17]. Two individual experiments were carried out in duplicate. Membrane disruption (%) was calculated from the kinetics measurements of the treated relative to the untreated cultures [17].

### 2.5. Gene Expression

The influence of pinocembrin on gene expression in *C. jejuni* NCTC 11168 Δ*cmeR* was evaluated using DNA microarray analysis and confirmed using quantitative real-time (qRT)-PCR, as previously described [17]. Overnight log-phase cultures incubated for 16 h at 42 °C and under a microaerophilic atmosphere were adjusted to OD_600_ of 0.2 in Mueller-Hinton broth using a spectrophotometer (Smart Spec; Bio-Rad, Hercules, CA, USA). These cultures were then treated with 16 µg/mL pinocembrin (i.e., 0.25 × MIC). DMSO (0.048%) was added as solvent to the untreated samples. These cultures were treated microaerobically for 2 h at 42 °C, with shaking. The experiments were carried out as three biological replicates. After the treatment, the RNA Protect Bacteria reagent (Qiagen, Maryland, USA) was added to the cultures to prevent mRNA degradation. Total RNA was isolated using RNeasy mini kits (Qiagen), and further treated using Ambion Turbo DNA-free kits (Invitrogen, Carlsbad, CA, USA), for removal of any contaminating DNA.

Quantitative real-time PCR was performed using a RT-PCR detection system (ABI 7500; Applied Biosystems, Thermo Scientific, Waltham, MA, USA) with the universal KAPA SYBR One-Step qRT-PCR kits (Kapa Biosystems, Boston, MA, USA). The qRT-PCR primers (Supplementary Table S1) were designed using the Primer3 online tool. A standard quantification curve was generated for each RNA template using 10-fold serial dilutions from 25 ng/µL to 0.0025 ng/µL. Quantitation was performed in 15 µL reactions with three technical replicates of three biological replicates, using the following program: 10 min at 50 °C; 5 min at 60 °C; 3 min at 95 °C; and 40 cycles of 10 s at 95 °C and 30 s at 58 °C. The melt curve analysis was performed after the amplification. The 16S rRNA gene served as an internal normalization standard for calculation of the relative fold-changes in gene expression, using the comparative threshold cycle method (ΔΔ*C*t) [18].

### 2.6. Intracellular Oxidation

The level of intracellular reactive oxygen species (ROS) as a result of treatments with a sub-inhibitory concentration of pinocembrin of 16 µg/mL (0.25 × MIC) was measured as described previously [14].

### 2.7. Oxidative Stress Reduction by Iron Chelation with 2,2-Dipyridyl

*Campylobacter jejuni* 11168∆*cmeR* suspensions (OD_600_, 0.2) were prepared in phosphate-buffered saline and then treated with pinocembrin (64 µg/mL), 2,2-dipyridyl (78 µg/mL) and pinocembrin and 2,2-dipyridyl combined [19]. An untreated *Campylobacter* culture was used as the control. The cultures were incubated for 24 h at 42 °C under microaerobic conditions. The experiment was carried out in triplicate, with sampling at 2, 6, and 24 h. The data are presented as log CFU/mL.

### 2.8. Motility Assay on Soft Agar

For the motility assay, *C. jejuni* NCTC 11168 and *C. jejuni* NCTC 11168 Δ*cmeR* were grown overnight on Mueller-Hinton agar and then re-suspended in phosphate-buffered saline to OD_600_ 0.2. Pinocembrin was added to the *Campylobacter* cultures at 32 µg/mL (0.5 × MIC) and an untreated *Campylobacter* culture was used as the control. These cultures were incubated for 2 h at 42 °C under microaerobic conditions. One microliter of the bacterial suspension was stabbed into 0.5% Mueller-Hinton agar and incubated for 48 h at 42 °C under microaerobic conditions. Motility was defined as the diameters of the colonies, with two independent experiments carried out in triplicate.

### 2.9. In-Vivo Testing of Campylobacter Virulence Potential

These experiments used *C. jejuni* NCTC 11168 and *C. jejuni* NCTC 11168 Δ*cmeR* as controls, and the same *C. jejuni* strains pre-treated with 32 µg/mL (0.5 × MIC) pinocembrin for 2 h at 42 °C under microaerobic conditions. The strains were grown overnight on Mueller-Hinton agar and re-suspended in phosphate-buffered saline to OD_600_ 0.2. In vivo experiments were performed in BALB/c mice according to ethical clearance No. 2170-24-01-3-07-02 and as previously described [19].

### 2.10. Statistical Analysis

Statistical analyses were carried out for the antimicrobial assays, intracellular oxidation, oxidative stress reduction by iron chelation, and intracellular energy metabolism by one-way ANOVA using SPSS Statistics 21 (IBM Corp., Armonk, NY, USA). Statistical analysis of the microarray results was carried out in R, using the LIMMA package [20].

## 3. Results

### 3.1. Anti-Campylobacter Activity of Pinocembrin, and the Resistance Mechanism

To evaluate the anti-*Campylobacter* activity of pinocembrin and the role of active efflux in *Campylobacter* resistance against pinocembrin, we determined the MICs for the wild-type *C. jejuni* NCTC 11168 and the three knockout mutants of *C. jejuni* NCTC 11168, with the defective efflux transporter genes *cmeB* and *cmeF* and transcriptional repressor *cmeR.* The antimicrobial activity of pinocembrin was moderate against *C. jejuni* NCTC 11168 and weaker compared to the antibiotics ciprofloxacin (MIC 0.062 µg/mL) and erythromycin (MIC 0.250 µg/mL), with a MIC of 64 µg/mL. When tested against the efflux pump mutants, the greatest changes in the susceptibility to pinocembrin were seen in the *cmeB* gene mutant, with decreased MICs for pinocembrin of 4-fold (Table 1). With the *cmeF* and *cmeR* mutants, no MIC changes were seen (Table 1, Supplementary Table S2), as compared with the wild-type *C. jejuni* NCTC 11168.

### 3.2. Campylobacter Fitness under Pinocembrin Treatment

**Figure 1:**
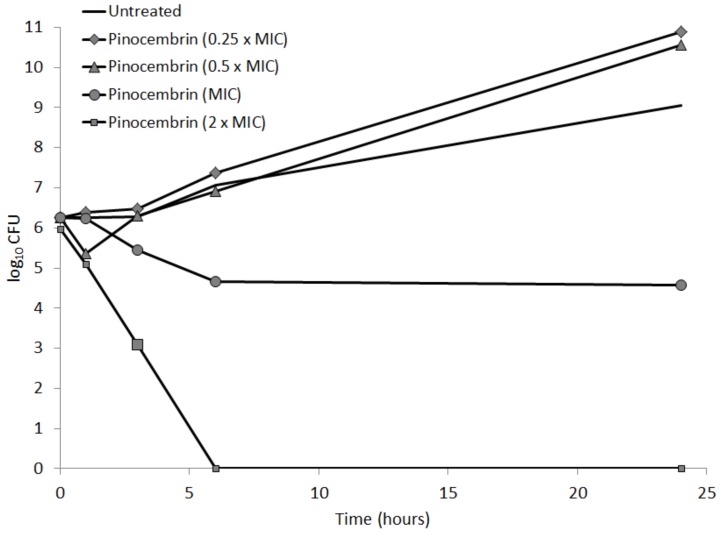
Time-kill kinetics of pinocembrin on *C. jejuni* NCTC 11168ΔcmeR at the sub-inhibitory concentrations of 16 µg/mL (0.25 × MIC) and 32 µg/mL (0.5 × MIC), the inhibitory concentration of 64 µg/mL (MIC), and the supra-inhibitory concentration of 128 µg/mL (2 × MIC), as indicated.

The antimicrobial activity of pinocembrin on *C. jejuni* NCTC 11168 Δ*cmeR* was further tested according to the time-kill kinetics of pinocembrin at the sub-inhibitory concentrations of 16 µg/mL (0.25 × MIC) and 32 µg/mL (0.5 × MIC), at the MIC of 64 µg/mL, and at the supra-inhibitory concentration of 128 µg/mL (2 × MIC). The survival of the Δ*cmeR* mutant was significantly decreased by over 4 log units at the MIC after 24 h of incubation at 42 °C, in comparison to the untreated control, as seen from the growth curves shown in Figure 1. With the treatment of the double MIC pinocembrin, the bactericidal effects were seen already after 1 h of exposure of *C. jejuni* to pinocembrin, with the viability decreased by over 1 log unit; after 6 h, all of the *C. jejuni* were dead (Figure 1). Interestingly, the viability of the Δ*cmeR* mutant increased by 1 log unit in the presence of both of the sub-inhibitory pinocembrin concentrations, as seen from the kinetics of the inactivation curves over 24 h (Figure 1).

### 3.3. Alterations of Campylobacter Membrane Integrity by Pinocembrin

**Figure 2:**
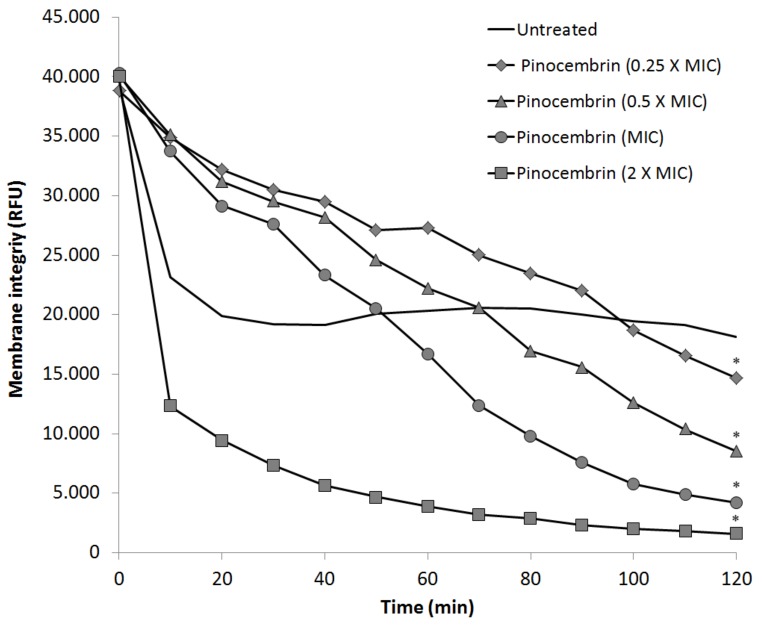
Influence of pinocembrin on membrane integrity of *C. jejuni* NCTC 11168ΔcmeR after 2-h treatment at the sub-inhibitory concentrations of 16 µg/mL (0.25 × MIC) and 32 µg/mL (0.5 × MIC), at 64 µg/mL (MIC), and at the supra-inhibitory concentration of 128 µg/mL (2 × MIC), as indicated. * *p* < 0.05.

The influence of pinocembrin on *C. jejuni* NCTC 11168 Δ*cmeR* membrane integrity was evaluated at the sub-inhibitory concentrations of 16 µg/mL (0.25 × MIC) and 32 µg/mL (0.5 × MIC), at the MIC of 64 µg/mL, and at the supra-inhibitory concentration of 128 µg/mL (2 × MIC). As expected, the highest pinocembrin (i.e., supra-inhibitory concentration) had large effects, with disruption of membrane integrity almost complete within 2 h (*p* < 0.05). At the MIC of pinocembrin, the membrane permeability was increased by 77% compared to the untreated culture (*p* < 0.05). All of the sub-inhibitory pinocembrin concentrations significantly altered membrane permeability in comparison to the untreated control (Figure 2).

### 3.4. Changes in Campylobacter Gene Expression by Pinocembrin

To define other possible modes of pinocembrin activity in *Campylobacter*, we evaluated the *Campylobacter* responses to the sub-inhibitory pinocembrin concentration of 16 µg/mL (0.25 × MIC). This treatment was expected to up-regulate the expression of genes in key pathways that are targeted by pinocembrin. After a 2-h treatment, 70 genes of NCTC 11168 Δ*cmeR* were differentially transcribed (with cut-off set at ≥2-fold difference; Table 2). Of these, 44 were up-regulated and 26 were down-regulated. The functional classification of these differentially expressed genes revealed that they were mainly involved in cell processes (23 genes), macromolecule metabolism (19 genes), and small molecule metabolism (16 genes).

The up-regulated genes after pinocembrin treatment were spread across 12 of the functional categories (Table 2, Supplementary Table S3), with most involved in small molecule metabolism (11) and macromolecule metabolism (10). For the small molecule metabolism, these genes were mostly involved in: energy metabolism (electron transport, aerobic metabolism); encoding of NADH dehydrogenase I (*nuoN, nuoK, nuoL, nuoH*); biosynthesis of co-cofactors, prosthetic groups, and carriers (molybdopterin, thiredoxin *trxA*, thiamine *moeB*); and amino-acid biosynthesis (serine family) and cysteine synthase (*cysM*). The *cj1153* gene was previously identified as a putative periplasmic cytochrome C that is involved in energy metabolism and was strongly up-regulated in this study (4.16-fold). The up-regulated genes for macromolecule metabolism were involved in the cell envelope, inside the groups of: membranes, lipoproteins, and porins; surface polysaccharides, lipopolysaccharides, and antigens (*pglJ*); surface structures with several flagellum genes (*fliK, flaC, flgE2, flaA, flgB, flgD*); and for a miscellaneous periplasmic protein (*p19*). Among these, the serine protease *htrA* was also expressed, with a function in degradation of macromolecules, such as proteins, peptides, and glycopeptides. Under the broad regulatory functions, two genes were up-regulated in signal transduction processes. Interestingly, the most strongly up-regulated genes (*Cj414*, 9.04-fold; *Cj415*, 7.78-fold; *Cj1338*, 3.73-fold) showed miscellaneous functions (8 genes) and unknown functions (Table 2, Supplementary Table S3).

On the other hand, under this pinocembrin treatment, the down-regulated genes were scattered across eight functional categories (Table 2, Supplementary Table S3). Similarly, most of these genes were involved in cell processes (11), including transport/ binding proteins (amino acids and amines, carbohydrates, organic acids, alcohols) and pathogenicity. *Cj0864* and *Cj1358c* were the most down-regulated genes, which encode a putative periplasmic protein (3.14-fold reduction), and a *nrfH* putative periplasmic cytochrome C (2.73-fold reduction), respectively. Among the genes involved in macromolecule metabolism (9 genes), five ribosomal proteins were down-regulated (*rpsD, rpsK, rpsM, rpsA*, *rpmG*), with two of these at higher levels (*rpsA*, *rpmG*: from 2.74-fold reduction to 2.87-fold reduction). Together with rRNA methylase (*cstA*) and transcription anti-termination protein (*nusG*), these genes are involved in synthesis and modification of macromolecules: both ribosomal and RNA synthesis. Two membrane proteins belong to the group of the cell envelope. Five genes with functions in small molecule metabolism are involved in energy metabolism, mostly in electron transport (*nrfG, nrfA*, iron Sulphur protein). Interestingly, the highly down-regulated aspartate ammonia–lyse protein (*aspA*) (3.05-fold reduction) is involved in central intermediary metabolism, with a general function. Three down-regulated proteins show other functions (2 genes) or miscellaneous functions (1 gene).

With qRT-PCR, up-regulation was confirmed for the superoxide dismutase (Fe) (*sodB*) gene and the putative flagellar hook assembly protein (*flgD)*, and down-regulation was confirmed for three genes, namely the 30S ribosomal protein S4 (*rpsD*), carbon starvation protein A homologue with putative function as an integral membrane protein (*cstA*), and putative periplasmic cytochrome C (*nrfH*). These three genes (*cstA*, *aspA*, *nrfH)* have important functions in macromolecule metabolism, small molecule metabolism, and cell processes, respectively, and they were strongly down-regulated by more than 2.5-fold reduction.

### 3.5. Intracellular ROS Formation

To confirm that oxidative stress was one of the key mechanisms of the pinocembrin antimicrobial activity, as suggested by the gene expression analyses, the intracellular levels of ROS were determined after treatment of *C. jejuni* NCTC 11168 Δ*cmeR* with 0.25 × MIC pinocembrin. The results did not confirm oxidative stress as one of the key antimicrobial mechanisms. The differences in intracellular levels of ROS between the treated and untreated cultures were not significant (*p* = 0.266) (Supplementary Figure S1).

### 3.6. Inhibition of Pinocembrin Activity with Fe Chelator 2,2-Dipyridyl

**Figure 3:**
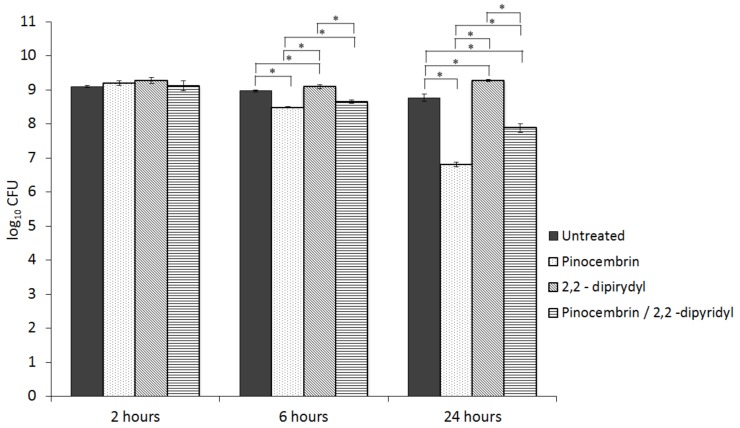
Inhibition of the pinocembrin (64 µg/mL; MIC) antibacterial activity on *C. jejuni* NCTC 11168ΔcmeR with the iron chelator 2,2-dipyridyl (78 µg/mL), as indicated. *, *p* < 0.05.

To examine the influence of pinocembrin on hydroxyl radical formation, we tested the viability of the *C. jejuni* 11168 Δ*cmeR* cultures under treatment with pinocembrin and 2,2-dipyridyl. 2,2-Dipyridyl is an iron chelator that can reduce harmful hydroxyl radicals generated in the Fenton reaction [22]. We found a significant influence of 2,2-dipyridyl on the pinocembrin action, especially after 24 h of treatment. The viabilities of the *C. jejuni* cultures treated with the combination of pinocembrin at the inhibitory concentration of 64 µg/mL plus 2,2-dipyridyl were significantly (*p* < 0.05) higher compared to those treated with the pinocembrin alone. Pinocembrin decreased *C. jejuni* NCTC 11168Δ*cmeR* viability by 2 log units after 24 h, which was decreased to 1 log unit with the addition of 2,2-dipyridyl. Treatment with 2,2-dipyridyl alone increased the viability by 0.5 log unit compared to the untreated *C. jejuni* cultures (Figure 3).

### 3.7. Modulation of Campylobacter Motility

The influence of pinocembrin on the motility of *C. jejuni* NCTC 11168Δ*cmeR* was measured on soft agar, as compared to untreated cells. After 2-h treatment with the sub-inhibitory pinocembrin concentration of 0.5 × MIC, the *C. jejuni* motility increased significantly. The diameter of the untreated *C. jejuni* cultures was 21.2 ± 1.6 mm, whereas after the pinocembrin treatment, this was 31.0 ± 1.9 mm (Table 3).

### 3.8. Modulation of Campylobacter Virulence under Pinocembrin Treatment

**Figure 4:**
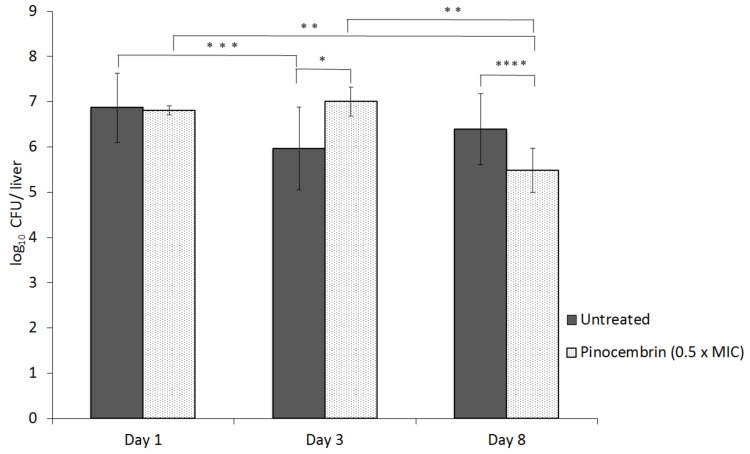
Influence of pinocembrin on *C. jejuni* NCTC 11168Δ*cmeR* in the livers of BALB/c mice. The mice were intravenously infected with untreated *C. jejuni* NCTC 11168Δ*cmeR* or *C. jejuni* NCTC 11168Δ*cmeR* treated with pinocembrin at the sub-inhibitory concentration of 32 µg/mL (0.5 × MIC), as indicated. Data are means ±standard deviation of *C. jejuni* colony-forming units (CFU)/liver. *, *p* = 0.006, **, *p* = 0.012, ***, *p* = 0.018 and ****, *p* = 0.024.

The influence of the sub-inhibitory pinocembrin concentration (32 µg/mL; 0.5 × MIC) on *C. jejuni* virulence was tested in a murine model. Four groups of BALB/c mice were intravenously infected with *C. jejuni* NCTC 11168 wild-type and the *C. jejuni* NCTC 11168 Δ*cmeR* mutant, as either untreated or pre-treated with the sub-inhibitory concentration of pinocembrin. After an initial and significant rise in the number of *C. jejuni* isolated for the third day post-infection, at eight days post-infection, the number of *C. jejuni* recovered from the mice livers in the pinocembrin-treated group diminished significantly in comparison to the untreated control (Figure 4).

## 4. Discussion

We have demonstrated antimicrobial activity of pinocembrin against *C. jejuni* and indicated possible mechanisms of its action. Specific intracellular sites of action and derivative targets would need to be further investigated to fully understand the mechanism of the mode of action of pinocembrin. Pinocembrin can potentially be used for medical applications or to serve as a chemical template for the design, synthesis and semi-synthesis of new substances for treatment of human *Campylobacter* infections [5,7].

In the present study, we first characterized the anti-*Campylobacter* activity of pinocembrin and defined the specific roles of the CmeABC and CmeDEF efflux pumps in resistance to pinocembrin. The 4-fold decrease in MIC of the mutant defective in *cmeB*, compared to wild-type, indicates possible involvement of the CmeABC efflux pump in the resistance against pinocembrin. The disruption of *cmeF* and *cmeR* did not have any effects on the MIC, which indicated that the CmeDEF efflux pump is not likely to be involved in *Campylobacter* protection against pinocembrin when CmeABC functions normally. These data are consistent with a previous study of Klančnik et al. [9], where the CmeABC efflux pump was shown to be an important resistance mechanism against plant extracts and phenolic compounds in *C. jejuni*. Therefore, further experiments were carried out on a null mutant in a *cmeABC* transcriptional repressor (Δ*cmeR*) that models an intrinsically resistant strain.

To determine the kinetics of pinocembrin on *C. jejuni* NCTC 11168 Δ*cmeR*, growth was tested at sub-inhibitory, inhibitory, and supra-inhibitory pinocembrin concentrations. These data confirmed pinocembrin as a bacteriostatic antimicrobial that sufficiently prevents growth at the MIC and significantly decreases viability at a supra-inhibitory concentration. Conversely, sub-inhibitory concentrations enhanced bacterial fitness.

Antibiotics targeting intracellular processes must penetrate the bacterial cell envelope. This applies in particular to the outer membrane of gram-negative bacteria, as this provides a formidable barrier [23]. We have shown here that pinocembrin affects the membrane integrity at inhibitory and sub-inhibitory concentrations, and that the effects observed are concentration dependent. As pinocembrin can increase membrane permeability and also act as an antimicrobial, it might be suitable for use in combination with other antimicrobials with low membrane permeability, to achieve synergistic effects. Sub-inhibitory concentrations of pinocembrin only slightly modulated *Campylobacter* membrane permeability and thus we can conclude that at sub-inhibitory concentrations, pinocembrin does not significantly decrease cell viability, but promotes bacterial growth and increases bacterial fitness. The up-regulation of 11 genes and down-regulation of 5 genes after exposure of *C. jejuni* to pinocembrin might indicate that pinocembrin treatment induces stress responses in *Campylobacter* cells. Additionally, the higher number of up-regulated genes involved in energy metabolism suggests stress responses [24]. Further, nine up-regulated genes involved in cell envelope synthesis indicate possible envelope stress responses [25].

These findings led us to investigate the effects of a sub-inhibitory concentration of pinocembrin on gene expression in *C. jejuni* NCTC 11168 Δ*cmeR* using microarrays and qRT-PCR. The most significantly up-regulated genes (>7-fold) were putative oxidoreductase subunits of unknown function (*Cj0414*, *Cj0415*). These two co-transcribed genes encode the two components of gluconate-oxidizing oxidoreductase, which is predicted to be localized in the periplasm, to peripherally associate with the cytoplasmic membrane, and to transfer electrons to periplasmic cytochrome c [26]. Up-regulation of these genes was seen also in response to acid shock, but it is not clear how this activity would contribute to the *C. jejuni* acid-shock response [27]. The changed transcription of a larger number of important genes points to a wider systemic response of *C. jejuni* in the presence of pinocembrin. We note here the significance of molybdoproteins Cj0725c, Cj0379c, and Cj1046-*moeB,* as these are also important in competition with the host microbiota. Moreover, for the genes involved in respiratory functions, as for the putative periplasmic cytochrome C (*cj1153*), which is important in defense against H_2_O_2_ through the reduction and detoxification of exogenous H_2_O_2_ [28,29]. Similarly, for the C551 peroxidase Cj0358, as one of the most down-regulated genes (2.89-fold reduction), which confirms that it is not significant in the resistance of *C. jejuni* against H_2_O_2_ [28,30]. On the other hand, two periplasmic cytochrome C proteins *nrfA* (Cj1357c) and *nrfH* (Cj1358c), and the putative ferredoxin *napG* (Cj0781; known as periplasmic located Nap-type nitrate reductase) were down-regulated in this study, which will disable nitrate as an electron acceptor in *C. jejuni* and further the reduction of nitrate to nitrite [30,31].

Up-regulation of the oxidative stress response genes *sodB, ahpC,* and *tpx* observed in the microarray study here indicates that pinocembrin treatment generates toxic ROS, like hydroxyl (^·^OH) and superoxide (O^−^_2_) radicals, as well as hydrogen peroxide (H_2_O_2_). Increased ROS causes damage to DNA, membranes, and proteins in the bacterial cell [32]. Consequently, a variety of ROS-detoxifying enzymes were expressed at the highest levels, which included the alkyl hydroxide reductase AhpC [33], superoxide dismutase SodB [34], and the thiolperoxidases Tpx [31,35]. The expression of oxidative stress defense genes in *C. jejuni* (e.g., *dps, sodB, trxB*, *ahpC*) was shown to increase also by exposure to HCl or acetic acid [36,37], which links the oxidative stress response mechanism and the acid-shock response in *C. jejuni* [27]. Additionally, the serine protease *htrA* (Cj1228c), required for heat and oxygen tolerance [38], was up-regulated, which indicated that it might be important for protection against pinocembrin treatment. As is known, these genes are also important to counteract oxidative and other stresses for optimal interactions with human epithelial cells, and during the host colonization processes of *C. jejuni* [38,39,40,41]. Recently, Kendall et al. [42] indicated that the OORC subunit of 2-oxoglutarate:acceptor *oorC* is one of the important genes involved in oxygen tolerance of *C. jejuni*. This gene (*oorC;* cj0538) was also up-regulated by pinocembrin.

This led us to investigate the phenotypic effects of pinocembrin on intracellular ROS formation and the decrease in hydroxyl radical formation by an iron chelator, which might explain the pinocembrin killing mechanism. Increased ROS levels in cells might indicate that oxidation occurs and antioxidant defense systems are a target of pinocembrin action. Previous studies of pinocembrin have shown its antioxidative activity in vitro as well as in vivo [43], while others have reported that it can cause oxidative damage in the presence of transition metal ions [44]. The underlying mechanism is thus relatively complex and no significantly decreased intracellular oxidation was observed in the present study. This might have been due to the mild oxidative stress caused by pinocembrin, rather than to its antioxidative activity. According to the results of our transcriptional study, weak oxidative stress induces the expression of oxidative stress defense genes. The higher levels of defensive enzymes can therefore efficiently neutralize ROS, which provides a protective effect that is reflected as ROS inhibition and time-kill kinetics. Furthermore, we confirmed the involvement of oxidative stress in the pinocembrin antibacterial activity by using 2,2-dipyridyl, an iron chelator that is involved in decreasing hydroxyl radical formation in the Fenton reaction When the *C. jejuni* cultures were treated with the combination of pinocembrin and 2,2-dipyridyl, the *C. jejuni* numbers compared to the treatment with only pinocembrin were halved. However, 2,2-dipyridyl did not counteract the effects of pinocembrin completely, which confirmed that oxidative stress is only part of the mechanism of pinocembrin. The involvements of other mechanisms together with oxidative stress, such as membrane disruption, are key mechanisms in the antimicrobial activity of pinocembrin.

It is known that as well as serine, proline, and glutamate catabolism, aspartate is crucial for the metabolic fitness of *C. jejuni* [45]. In the present study, down-regulated expression was seen for the C4-dicarboxylate transporter gene *dcuB (Cj0671)* and the aspartase gene *aspA* (Cj0087), both of which are important for persistence of *C. jejuni* in its host [44,45]. Down-regulation with pinocembrin was also seen for the putative HAMP-containing membrane protein Cj0952c, which affects chemotactic behavior towards formic acid and is important for invasion of host cells [45], the *Cj0073c* gene, which is important for NAD-independent respiratory lactate dehydrogenase activity, and the *Cj0903c* gene, which is a gene for putative amino-acid transporters with homology to members of the sodium:alanine symporter family with unknown substrate specificity [45]. The down-regulation of ribosomal proteins observed with pinocembrin treatment correlated with the lower levels required for protein synthesis and biosynthesis of amino acids, purines and fatty acids during the extended intracellular persistence inside the *Campylobacter*-containing vacuole. Further, several protein-degrading and peptide-degrading proteins and transporters involved in the uptake of amino acids, phosphate, and metals showed lower levels 20 h post-infection [45]. In contrast, a transporter involved in the uptake of iron *cfbpA* (Cj0175c) was up-regulated under pinocembrin.

The C. jejuni bipolar flagellum is particularly important for its motility. Interestingly, under pinocembrin treatment, the up-regulated proteins are involved in all three conserved parts of the bipolar flagellum; namely, the basal body, hook, and filament. In the present study, flagellin *flaA* (*Cj1139c*) was up-regulated, which is one of two flagellins (FlaA, FlaB) of the flagellar filament [28,46]. The flagellar channel serves as a non-conventional type III secretion system that is necessary for the export of the adhesive protein FlaC that influences invasion and interactions with the host environment [46]. This flagellin gene *flaC* (*Cj0720c*) was up-regulated by 2.22-fold under pinocembrin treatment. Similarly, there was up-regulation of some flagellar hook protein regulatory genes: *fliK* (*Cj0041*), which encodes a hook length control and export specificity switch, and *flgD* (*Cj0042*) and *flgE* (*Cj1729c*), which have important roles in motility, flagellar filament assembly, hook assembly, and regulation of flagellin synthesis and secretion [47]. The basal body constitutes a major portion of the flagellar organelle and consists of a number of rings that are mounted on a central rod [46]. The gene *flgB* (*Cj0528c*) encodes the flagellar basal body rod protein FlgB and this was up-regulated 2.15-fold under pinocembrin treatment. Furthermore, Gaynor et al. [48] reported that protein misfolding and degradation are consistent with a stress-related response to misdirection of the assembly of the flagellum.

In *Campylobacter*, flagellar biosynthesis is co-regulated with virulence [46], making motility a major virulence factor in its infection and pathogenesis [49,50]. We confirmed the data from the transcriptomic analysis at a phenotypic level, with significantly increased *Campylobacter* motility after pinocembrin treatment. This confirmed the importance of up-regulated genes involved in flagellar assembly and motility after pinocembrin treatment. Up-regulation of flagellar genes and increased motility in *Campylobacter* have been linked to acid-shock exposure [51]. In agreement with this, in acid-shocked *Campylobacter* there is increased expression of flagellar biosynthetic genes, as genes involved in oxidative and general stress responses. This response presents a problem, as acid shock in *Campylobacter* not only increases their motility, but also increases their passing through the acidic gastric environment and their ability to invade the m-IC_cl2_ mouse small intestine crypt cells [51].

We noted that under pinocembrin treatment, *C. jejuni* Δ*cmeR* showed up-regulation of the expression of heat-shock, acid-shock, and oxygen stress response genes. It is known that the flagellum (basal body, hook, and filament portions) and also the heat-shock response in *C. jejuni* influences colonization of a chicken host [40,52,53]. In the present study, we investigated the systemic infection of BALB/c mice with pinocembrin-treated *C. jejuni* Δ*cmeR*. On the third day after infection, the number of pinocembrin-treated *Campylobacter* was higher than that in the control group, which can be explained by the up-regulation of motility-associated proteins, including *flaA*, *flaC,* and *fliK*, and stress-response genes that contribute to the resistance of *C. jejuni* in the host environment. Despite this initial increase, there were later reductions in the bacterial numbers in the liver of the host, with a drop in numbers by the eighth day post-infection, which makes the advantage at day three a temporary phenomenon. This suggests increased sensitivity of pinocembrin-treated *Campylobacter* to the host immune response. Consequently, pinocembrin-treated *C. jejuni* cells were less likely to persist in the host.

## 5. Conclusions

In conclusion, pinocembrin, a compound naturally occurring in foods such as honey, shows antimicrobial activity towards *Campylobacter* through reduction of membrane integrity. However, this activity is lost at a sub-inhibitory concentration of pinocembrin, which also, in contrast, promotes bacterial growth and increases bacterial fitness. The subinhibitory concentration of pinocembrin modified transcription of *Campylobacter* genes involved in cell-wall synthesis, heat-shock, acid-shock, and oxidative-stress, which is likely to result in improved fitness, membrane integrity and oxidative stress defense.

## Figures and Tables

**Table 1:** Antimicrobial activities of ciprofloxacin, erythromycin, and pinocembrin against *C. jejuni* NCTC 11168 and its efflux pump knockout mutant strains that lack functional genes for efflux pumps CmeABC (Δ*cmeB*) and CmeDEF (Δ*cmeF*), and the efflux pump repressor CmeR (Δ*cmeR*), presented as minimal inhibitory concentration (MIC) in µg/mL.

*Strain*	MIC (µg/mL)		
	Ciprofloxacin	Erythromycin	Pinocembrin
*C. jejuni* NCTC 11168	0.062	0.25	64
*C. jejuni* NCTC 11168Δ*cmeB*	0.016	0.062	16
*C. jejuni* NCTC 11168Δ*cmeF*	0.125	0.25	64
*C. jejuni* NCTC 11168Δ*cmeR*	0.25	0.5	64

**Table 2:** Differentially expressed genes after treatment of *C. jejuni* 11168 Δ*cmeR* with pinocembrin (16 µg/mL, as 0.25 × MIC).

Functional Classification of Predicted *C. jejuni* Genes *	Differentially Expressed Genes (n)	Up-Regulated Genes (n)	Down-Regulated Genes (n)
**1 Small molecule metabolism**	**16**	**11**	**5**
*1.B Energy metabolism*		6	4
*1.G Biosynthesis of cofactors, prosthetic groups and carriers*		4	0
*1.C Central intermediary metabolism*		0	1
*1.D Amino acid biosynthesis*		1	0
**2 Broad regulatory functions**	**2**	**2**	**0**
*2.1 Signal transduction*		2	
**3 Macromolecule metabolism**	**20**	**10**	**9**
*3.A Synthesis and modification of macromolecules*		0	7
*3.B Degradation of macromolecules*		1	0
*3.C Cell envelope*		9	2
**4 Cell processes**	**23**	**12**	**11**
*4.A Transport/ binding proteins*		4	2
*4.E Protein and peptide secretion*		1	0
*4.G Detoxification*		3	0
*4.I Pathogenicity*		4	7
**5 Other**	**3**	**1**	**2**
*5.H Conserved hypothetical proteins*		1	2
**6. Miscellaneous**	**9**	**8**	**1**
**Total**	**73**		

* Classification is based on that used for *Escherichia coli* by Riley, M. and Labedan, B. in *Escherichia coli and Salmonella* (ed. Neidhardt, F.C.) 2118–2202 (ASM, Washington, 1996) [21].

**Table 3:** Influence of pinocembrin on *C. jejuni* NCTC 11168ΔcmeR motility on soft agar after 2-h treatment at the sub-inhibitory concentrations of 16 µg/mL (0.25 × MIC) and 32 µg/mL (0.5 × MIC), as indicated. Data are means ±standard deviation for the diameter measured on the agar plate.

*C. jejuni* NCTC 11168Δ*cmeR*	Diameter (mm)
Untreated	21.2 ± 1.57
Pinocembrin (0.25 × MIC)	19 ± 0.81
Pinocembrin (0. 5 × MIC)	31 ± 1.91 ^a^

^a^*p* < 0.05 (vs. untreated control).

## Supplementary Materials

The following are available online at https://www.mdpi.com/2076-2607/7/12/675/s1: Figure S1: Time-course of influence of sub-inhibitory pinocembrin (16 μg/mL; 0.25 × MIC) on intracellular reactive oxygen species in *Campylobacter jejuni* NCTC 11168*ΔcmeR*; Table S1: Primers of the genes for qRT-PCR confirmation used in this study; Table S2: Antimicrobial activity of ciprofloxacin (CIP; 5 μg), erythromycin (ERY 15 μg) and pinocembrin (Pc 100 μg, 50 μg, 25 μg, 12.5 μg) against *C. jejuni* NCTC 11168 and its efflux pump knockout mutant strains lacking the functional genes for efflux pumps CmeABC (*ΔcmeB*) and CmeDEF (*ΔcmeF*), and the efflux pump repressor CmeR (*ΔcmeR*), presented as inhibition zone diameters (mm); Table S3: Differentially expressed genes after treatment of *C. jejuni* 11168 *ΔcmeR* with pinocembrin (0.25 × MIC).

## References

[1] EFSA (2017). The European Union summary report on trends and sources of zoonoses, zoonotic agents and food-borne outbreaks in 2016. EFSA J..

[2] Nyati K.K., Nyati R. (2013). Role of *Campylobacter jejuni* infection in the pathogenesis of Guillain-Barré syndrome: an update. Biomed. Res. Int..

[3] ECDC (2018). Antimicrobial Resistance in Zoonotic Bacteria Still High in Humans, Animals and Food, Say ECDC and EFSA. European Centre for Disease Prevention and Control (ECDC). https://www.ecdc.europa.eu/en/news-events/antimicrobial-resistance-zoonotic-bacteria-still-high-humans-animals-and-food-say-ecdc.

[4] Friedman M. (2015). Antibiotic-resistant bacteria: prevalence in food and inactivation by food-compatible compounds and plant extracts. J. Agric. Food Chem..

[5] Lan X., Wang W., Li Q., Wang J. (2016). The natural flavonoid pinocembrin: Molecular targets and potential therapeutic applications. Mol. Neurobiol..

[6] Aiello F., Armentano B., Polerà N., Carullo G., Loizzo R.M., Bonesi M., Cappello M.S., Capobianco L., Tundis R. (2017). From vegetable waste to new agents for potential health applications: Antioxidant properties and effects of extracts, fractions and pinocembrin from Glycyrrhiza glabra *L. aerial* parts on viability of five human cancer cell lines. J. Agric. Food Chem..

[7] Rasul A., Millimouno F.M., Ali Eltayb W., Ali M., Li J., Li X. (2013). Pinocembrin: a novel natural compound with versatile pharmacological and biological activities. Biomed. Res. Int..

[8] Lin J., Overbye M.L., Zhang Q. (2002). CmeABC functions as a multidrug efflux system in *Campylobacter jejuni*. Antim. Agents Chemotherap..

[9] Klančnik A., Smole Možina S., Zhang Q. (2012). Anti-*Campylobacter* activities and resistance mechanisms of natural phenolic compounds in *Campylobacter*. PLoS ONE.

[10] Lin J., Akiba M., Sahin O., Zhang Q. (2005). CmeR functions as a transcriptional repressor for the multidrug efflux pump CmeABC in *Campylobacter jejuni*. Antim. Agents Chemotherap..

[11] Yang W., Zhang M., Zhou J., Pang L., Wang G., Hou F. (2017). The molecular mechanisms of ciprofloxacin resistance in clinical *Campylobacter jejuni* and their genotyping characteristics in Beijing, China. Foodborne Pathog. Dis..

[12] Zhang T., Cheng Y., Luo Q., Lu Q., Dong J., Zhang R., Wen G., Wang H., Luo L., Wang H. (2017). Correlation between *gyrA* and CmeR box polymorphism and fluoroquinolone resistance in *Campylobacter jejuni* isolates in China. Antimicrob. Agents Chemoth..

[13] Lekshmi M., Ammini P., Kumar S., Varela M.F. (2017). The food production environment and the development of antimicrobial resistance in human pathogens of animal origin. Microorganisms.

[14] Klančnik A., Guzej B., Hadolin Kolar M., Abramovič H., Smole Možina S. (2009). In-vitro antimicrobial and antioxidant activity of commercial rosemary extract formulations. J. Food Prot..

[15] EUCAST 2019 The European Committee on Antimicrobial Susceptibility Testing. Breakpoint tables for interpretation of MICs and zone diameters. Version 9.0, 2019. http://www.eucast.org/fileadmin/src/media/PDFs/EUCAST_files/Breakpoint_tables/v_9.0_Breakpoint_Tables.pdf.

[16] Klančnik A., Piskernik S., Jeršek B., Smole Možina S. (2010). Evaluation of diffusion and dilution methods to determine the antibacterial activity of plant extracts. J. Microbiol. Methods.

[17] Kovač J., Šimunović K., Wu Z., Klančnik A., Bucar F., Zhang Q. (2015). Smole Možina, S. Antibiotic resistance modulation and modes of action of (-)-α-pinene in *Campylobacter jejuni*. PLoS ONE.

[18] Livak K.J., Schmittgen T.D. (2001). Analysis of relative gene expression data using real-time quantitative PCR and the 2(-Delta Delta C(T)) method. Methods.

[19] Klančnik A., Vučković D., Plankl M., Abram M., Smole Možina S. (2013). *In-vivo* modulation of *Campylobacter jejuni* virulence in response to environmental stress. Foodborne Pathog. Dis..

[20] Wu Z., Sahin O., Shen Z., Liu P., Miller W.G., Zhang Q. (2013). Multi-omics approaches to deciphering a hypervirulent strain of *Campylobacter jejuni*. Genome Biol. Evol..

[21] Riley M., Labedan B., Neidhardt F.C., Curtiss R., Ingraham J.L., Lin E.C.C., Low K.B., Magasanik B., Reznikoff W.S., Riley M., Schaechter M., Umbarger H.E. (1996). Gene products: physiological functions and common ancestries. Escherichia coli and Salmonella: Cellular and Molecular Biology.

[22] Kohanski M.A., Dwyer D.J., Hayete B., Lawrence C.A., Collins J.J. (2007). A common mechanism of cellular death induced by bactericidal antibiotics. Cell.

[23] Delcour A.H. (2009). Outer membrane permeability and antibiotic resistance. Biochim. Biophys. Acta.

[24] Schwachtje J., Whitcomb S.J., Firmino A.A.P., Zuther E., Hincha D.K., Kopka K. (2019). Induced, Imprinted, and primed responses to changing environments: Does metabolism store and process information?. Front. Plant Sci..

[25] Hews C.L., Cho T., Rowley G., Raivio T.L. (2019). Maintaining integrity under stress: envelope stress response regulation of pathogenesis in gram-negative bacteria. Front. Cell. Infect. Microbiol..

[26] Pajaniappan M., Hall J.E., Cawthraw S.A., Newell D.G., Gaynor E.C., Fields J.A., Rathbun K.M., Agee W.A., Burns C.M., Hall S.J. (2008). A temperature-regulated *Campylobacter jejuni* gluconate dehydrogenase is involved in respiration-dependent energy conservation and chicken colonization. Mol. Microbiol..

[27] Reid A.N., Pandey R., Palyada K., Naikare H., Stintzi A. (2008). Identification of *Campylobacter jejuni* genes involved in the response to acidic pH and stomach transit. Appl. Environ. Microbiol..

[28] Hendrixsonm D.R., DiRitam V.J. (2004). Identification of *Campylobacter jejuni* genes involved in commensal colonization of the chick gastrointestinal tract. Mol. Microbiol..

[29] Sellars M.J., Hall S.J., Kelly D.J. (2002). Growth of *Campylobacter jejuni* supported by respiration of fumarate, nitrate, nitrite, trimethylamine-N-oxide, or dimethyl sulfoxide requires oxygen. J. Bacteriol..

[30] Bingham-Ramos L.K., Hendrixson D.R. (2008). Characterization of two putative cytochrome c peroxidases of *Campylobacter jejuni* involved in promoting commensal colonization of poultry. Infect. Immun..

[31] Kim J.-C., Oh E., Kim J., Jeon B. (2015). Regulation of oxidative stress resistance in *Campylobacter jejuni*, a microaerophilic foodborne pathogen. Front. Microbiol..

[32] Pittman M.S., Elvers K.T., Lee L., Jones M.A., Poole R.K., Park S.F., Kelly D.J. (2007). Growth of *Campylobacter jejuni* on nitrate and nitrite: electron transport to NapA and NrfA via NrfH and distinct roles for NrfA and the globin Cgb in protection against nitrosative stress. Mol. Microbiol..

[33] Baillon M.L., Van Vliet A.H., Ketley J.M., Constantinidou C., Penn C.W. (1999). An iron-regulated alkyl hydroperoxide reductase (AhpC) confers aerotolerance and oxidative stress resistance to the microaerophilic pathogen *Campylobacter jejuni*. J. Bacteriol..

[34] Pesci E.C., Cottle D.L., Pickett C.L. (1994). Genetic, enzymatic, and pathogenic studies of the iron superoxide dismutase of *Campylobacter jejuni*. Infect. Immun..

[35] Atack J.M., Kelly D.J. (2008). Contribution of the stereospecific methionine sulphoxide reductases MsrA and MsrB to oxidative and nitrosative stress resistance in the food-borne pathogen *Campylobacter jejuni*. Microbiology.

[36] Murphy C., Carroll C., Jordan K.N. (2003). Induction of an adaptive tolerance response in the foodborne pathogen, *Campylobacter jejuni*. FEMS Microbiol. Lett..

[37] Birk T., Wik M.T., Lametsch R., Knøchel S. (2012). Acid stress response and protein induction in *Campylobacter jejuni* isolates with different acid tolerance. BMC Microbiol..

[38] Brøndsted L., Andersen M.T., Parker M., Jørgensen K., Ingmer H. (2005). The HtrA protease of *Campylobacter jejuni* is required for heat and oxygen tolerance and for optimal interaction with human epithelial cells. Appl. Environ. Microbiol..

[39] Novik V., Hofreuter D., Galán J.E. (2010). Identification of *Campylobacter jejuni* genes involved in its interaction with epithelial cells. Infect. Immun..

[40] Palyada K., Sun Y.Q., Flint A., Butcher J., Naikare H., Stintzi A. (2009). Characterization of the oxidative stress stimulon and PerR regulon of *Campylobacter jejuni*. BMC Genom..

[41] Stintzi A., Marlow D., Palyada K., Naikare H., Panciera R., Whitworth L., Clarke C. (2005). Use of genome-wide expression profiling and mutagenesis to study the intestinal lifestyle of *Campylobacter jejuni*. Infect. Immun..

[42] Kendall J.J., Barrero-Tobon A.M., Hendrixson D.R., Kelly D.J. (2014). Hemerythrins in the microaerophilic bacterium *Campylobacter jejuni* help protect key iron-sulphur cluster enzymes from oxidative damage. Environ. Microbiol..

[43] Mavri A., Abramovič H., Polak T., Bertoncelj J., Jamnik P., Smole Možina S., Jeršek B. (2012). Chemical properties and antioxidant and antimicrobial activities of Slovenian propolis. Chem. Biodivers..

[44] Tsai Y.-C., Wang Y.-H., Liou C.-C., Lin Y.-C., Huang H., Liu Y.-C. (2012). Induction of oxidative DNA damage by flavonoids of propolis: its mechanism and implication about antioxidant capacity. Chem. Res. Toxicol..

[45] Hofreuter D. (2014). Defining the metabolic requirements for the growth and colonization capacity of *Campylobacter jejuni*. Front. Cell. Infect. Microbiol..

[46] Carrillo C.D., Taboada E., Nash J.H.E., Lanthier P., Kelly J., Lau P.C., Verhulp R., Mykytczuk O., Jonathan S., Findlay W.A. (2004). Genome-wide expression analyses of *Campylobacter jejuni* NCTC11168 reveals coordinate regulation of motility and virulence by *flhA*. J. Biol. Chem..

[47] Gaynor E.C., Cawthraw S., Manning G., MacKichan J.K., Falkow S., Newell D.G. (2004). The genome-sequenced variant of *Campylobacter jejuni* NCTC 11168 and the original clonal clinical isolate differ markedly in colonization, gene expression, and virulence-associated phenotypes. J. Bacteriol..

[48] Gaynor E.C., Wells D.H., MacKichan J.K., Falkow S. (2005). The *Campylobacter jejuni* stringent response controls specific stress survival and virulence-associated phenotypes. Mol. Microbiol..

[49] Guerry P., Poly F., Riddle M., Maue A.C., Chen Y.H., Monteiro M.A. (2012). *Campylobacter* polysaccharide capsules: virulence and vaccines. Front. Cell Infect. Microbiol..

[50] Stintzi C.M., King M., Haardt M., Armstrong G.D. (1995). *Campylobacter jejuni* motility and invasion of *Caco-2* cells. Infect. Immun..

[51] Le M.T., Porcelli I., Weight C.M., Gaskin D.J.H., Carding S.R., van Vliet A.H.M. (2012). Acid-shock of *Campylobacter jejuni* induces flagellar gene expression and host cell invasion. Eur. J. Microbiol. Immunol..

[52] Kalmokoff M., Lanthier P., Tremblay T.L., Foss M., Lau P.C., Sanders G., Austin J., Kelly J., Szymanski C.M. (2006). Proteomic analysis of *Campylobacter jejuni* 11168 biofilms reveals a role for the motility complex in biofilm formation. J. Bacteriol..

[53] Konkel M.E., Kim B.J., Klena J.D., Young C.R., Ziprin R. (1998). Characterization of the thermal stress response of *Campylobacter jejuni*. Infect. Immun..

