# Supplementary material for: The Anti-Campylobacter Activity and Mechanisms of Pinocembrin Action
Source: Microorganisms. 2019 Dec 10;7(12):675. doi: 10.3390/microorganisms7120675 (PMC6955772; doi:10.3390/microorganisms7120675)
Supplement: Supplementary file 1 [file microorganisms-07-00675-s001.pdf]

**Supplementary Materials - Figure**

**Figure S1.** Time-course of influence of sub-inhibitory pinocembrin (16 µg/mL; 0.25 × MIC) on intracellular reactive oxygen species in *Campylobacter jejuni* NCTC 11168ΔcmeR.

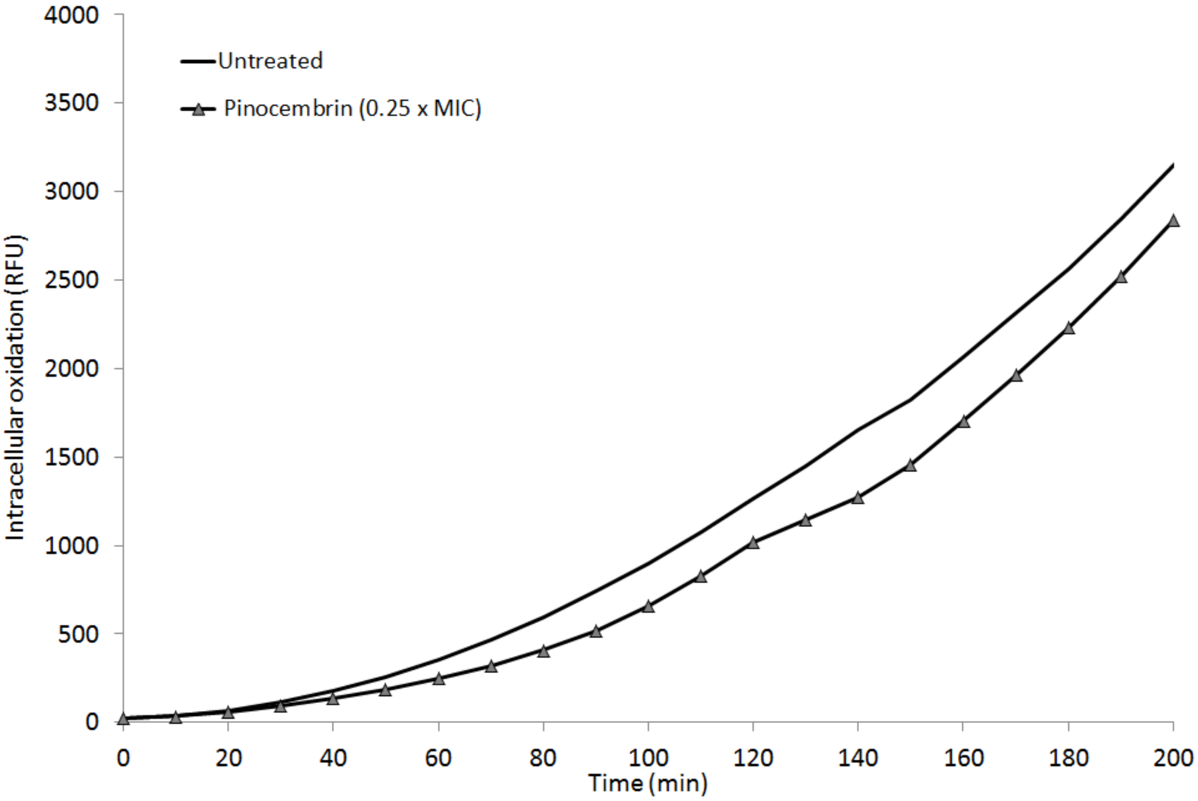


**Figure S1**

**Supplementary Materials - Tables**

**Table S1.** Primers of the genes for qRT-PCR confirmation used in this study.

| **Target** | **Forward primer (5’-3’)** | **Reverse primer (5’-3’)** |
|---|---|---|
| *nrfH* | AAG TCA AAG GCG AAC CAA GA | CCT AAA CCA CTT TGC GCT TT |
| *aspA* | TGG GGA ATT GGA AAT CTC TG | CCC TAA CAA AGC GAG GAA AA |
| *cstA* | GTT GCT ATG GTG GCG ATT TT | CCC CAT GGA GAT TCT GCT AA |
| *rpsD* | TGC TTG GGT TGA TGT TGA AA | CCT CAA CCG GAA TGA CAA CT |
| *nuoM* | TGG GCA CCTA AGG TTT ATG C | CAC ACT CGC ATC TGG AAA AA |
| *cfbpA* | AAG CAT GGG CTA AAG GTG TG | AAA TTT AGC TTC GCC TGC AA |
| *flaB* | TAG CGC AGG AAG TGG ATT TT | TAG CGT TTG CAA AAC CTG TG |
| *ahpC* | CTG CTC CAG CGG TAT TAG GA | TAC CGC TCC TTT TGG ACC TA |
| *flgD* | TAT GCA AAA ATG GCT GGA CA | TGA ACC GCT TCC TCC AGT AG |
| *sodB* | TGG TTC AGG ATG GTT TTG GT | TGG TGT AGC TGC GTT TGA AG |

**Table S2.** Antimicrobial activity of ciprofloxacin (CIP; 5 µg), erythromycin (ERY 15 µg) and pinocembrin (Pc 100 µg, 50 µg, 25 µg and 12.5 µg) against *C. jejuni* NCTC 11168 and its efflux pump knockout mutant strains lacking functional genes for efflux pumps CmeABC (Δ*cmeB*) and CmeDEF (Δ*cmeF*), and the efflux pump repressor CmeR (Δ*cmeR*), presented as inhibition zone diameter in mm.

| | | **Inhibition zone diameter (mm)** | | | | | |
|---|---|---|---|---|---|---|---|
| **Method** | **Strain** | **CIP** | **ERY** | **Pc 100** | **Pc 50** | **Pc 25** | **Pc 12.5** |
| | 11168 | 38 | 44 | 16 | 14 | 10 | 6 |
| Agar-well diffusion | Δ*cmeB* | 36 | 45 | 29 | 27 | 21 | 9 |
| (Well diameter 4 mm) | Δ*cmeF* | 36 | 41 | 14 | 12 | 8 | 6 |
| | Δ*cmeR* | 35 | 38 | 17 | 15 | 11 | 8 |
| | 11168 | 34 | 40 | 13 | 11 | 9 | 7* |
| Disc diffusion | Δ*cmeB* | 39 | 39 | 25 | 18 | 15 | 8 |
| (Disc diameter 7 mm) | Δ*cmeF* | 33 | 38 | 15 | 11 | 9 | 7* |
| | Δ*cmeR* | 33 | 35 | 14 | 12 | 10 | 7* |

*No inhibition zone

**Table S3.** Differentially expressed genes after treatment of *C. jejuni* 11168 Δ*cmeR* with pinocembrin (0.25 x MIC).

| **Gene ID and Functional Category** | | | ***P*-value** | ***Q*-value** | ***n*-Fold change** | |
|---|---|---|---|---|---|---|
| | | | | | **Microarray** | **qRT-PCR** |
| *Cj0414* | | putative oxidoreductase subunit | 0.0000 | 0.0000 | 9.04 | NT |
| *Cj0415* | | putative GMC oxidoreductase subunit | 0.0000 | 0.0000 | 7.48 | NT |
| *Cj0334* | *ahpC* | alkyl hydroperoxide reductase | 0.0000 | 0.0000 | 6.13 | 1.8 |
| *Cj1153* | | putative periplasmic cytochrome C | 0.0000 | 0.0003 | 4.16 | NT |
| *Cj0169* | *sodB* | superoxide dismutase (Fe) | 0.0000 | 0.0002 | 3.81 | 2.7 |
| *Cj1388* | | putative endoribonuclease L-PSP | 0.0008 | 0.0058 | 3.73 | NT |
| *Cj0779* | *tpx* | thiol peroxidase | 0.0000 | 0.0003 | 3.36 | NT |
| *Cj1506c* | | putative MCP-type signal transduction protein | 0.0000 | 0.0003 | 2.98 | NT |
| *Cj0420* | | putative periplasmic protein | 0.0001 | 0.0016 | 2.97 | NT |
| *Cj0239c* | | NifU protein homolog | 0.0000 | 0.0003 | 2.79 | NT |
| *Cj0236c* | | putative integral membrane protein | 0.0000 | 0.0008 | 2.76 | NT |
| *Cj0416* | | hypothetical protein | 0.0006 | 0.0051 | 2.76 | NT |
| *Cj0379c* | | putative molybdenum containing oxidoreductase | 0.0060 | 0.0181 | 2.75 | NT |
| *Cj1338c* | *flaB* | flagellin | 0.0001 | 0.0019 | 2.70 | 1.1 |
| *Cj0515* | | putative periplasmic protein | 0.0529 | 0.0735 | 2.65 | NT |
| *Cj0041* | *fliK* | putative flagellar hook-length control protein | 0.0050 | 0.0162 | 2.64 | NT |
| *Cj0987c* | | putative MFS (Major Facilitator Superfamily) | 0.0000 | 0.0004 | 2.51 | NT |

| | | | | | | |
|---|---|---|---|---|---|---|
| *Cj0378c* | | putative ferric reductase-like transmembrane | 0.0000 | 0.0009 | 2.48 | NT |
| *Cj0725c* | | molybdopterin biosynthesis protein | 0.0000 | 0.0009 | 2.43 | NT |
| *Cj0147c* | *trxA* | thioredoxin | 0.0053 | 0.0166 | 2.43 | NT |
| *Cj1224* | | putative iron-binding protein | 0.0011 | 0.0071 | 2.39 | NT |
| *Cj0998c* | | putative periplasmic protein | 0.0004 | 0.0040 | 2.38 | NT |
| *Cj1659* | *p19* | periplasmic protein p19 | 0.0131 | 0.0298 | 2.37 | NT |
| *Cj0175c* | *cfbpA* | putative iron-uptake ABC transport | 0.0012 | 0.0073 | 2.34 | 1.1 |
| *Cj0040* | | hypothetical protein | 0.0034 | 0.0130 | 2.24 | NT |
| *Cj1569c* | *nuoK* | NADH dehydrogenase I chain K | 0.0001 | 0.0018 | 2.24 | NT |
| *Cj0720c* | *flaC* | flagellin | 0.0016 | 0.0086 | 2.22 | NT |
| *Cj1566c* | *nuoN* | NADH dehydrogenase I chain N | 0.0000 | 0.0009 | 2.24 | NT |
| *Cj0417* | | hypothetical protein | 0.0025 | 0.0110 | 2.23 | NT |
| *Cj1729c* | *flgE2* | flagellar hook subunit protein | 0.0091 | 0.0237 | 2.20 | NT |
| *Cj1339c* | *flaA* | flagellin | 0.0004 | 0.0038 | 2.19 | NT |
| *Cj0528c* | *flgB* | flagellar basal-body rod protein | 0.0001 | 0.0018 | 2.15 | NT |
| *Cj0898* | | putative histidine triad (HIT) family protein | 0.1469 | 0.1453 | 2.13 | NT |
| *Cj0830* | | putative integral membrane protein | 0.0388 | 0.0601 | 2.12 | NT |
| *Cj1228c* | *htrA* | serine protease (protease DO) | 0.0001 | 0.0018 | 2.11 | NT |
| *Cj1127c* | *pglJ* | GalNAc transferase | 0.0000 | 0.0010 | 2.10 | NT |
| *Cj1046c* | *moeB* | putative molybdopterin biosynthesis protein | 0.0001 | 0.0020 | 2.08 | NT |
| *Cj1534c* | | putative bacterioferritin | 0.0138 | 0.0313 | 2.08 | NT |
| *Cj1626c* | | putative periplasmic protein | 0.0004 | 0.0041 | 2.06 | NT |

| | | | | | | |
|---|---|---|---|---|---|---|
| *Cj0912c* | *cysM* | cysteine synthase | 0.0019 | 0.0093 | 2.06 | NT |
| *Cj1572c* | *nuoH* | NADH dehydrogenase I chain H | 0.0165 | 0.0349 | 2.03 | NT |
| *Cj1568c* | *nuoL* | NADH dehydrogenase I chain L | 0.0004 | 0.0038 | 2.03 | NT |
| *Cj0538* | *oorC* | OORC subunit of 2-oxoglutarate:acceptor | 0.0002 | 0.0028 | 2.02 | NT |
| *Cj0440c* | | putative transcriptional regulator | 0.0001 | 0.0014 | 2.00 | NT |
| *Cj0042* | *flgD* | putative flagellar hook assembly protein | 0.0008 | 0.0059 | 1.97 | 2.5 |
| *Cj1567c* | *nuoM* | NADH dehydrogenase I chain M | 0.0032 | 0.0128 | -1.62 | -1.1 |
| *Cj1594* | *rpsD* | 30S ribosomal protein S4 | 0.0001 | 0.0020 | -1.76 | -3.2 |
| *Cj0781* | *napG* | putative ferredoxin | 0.0009 | 0.0065 | -2.02 | NT |
| *Cj0952c* | | putative HAMP containing membrane protein | 0.0035 | 0.0132 | -2.04 | NT |
| *Cj0903c* | | putative amino-acid transport protein | 0.0011 | 0.0069 | -2.05 | NT |
| *Cj0892c* | | putative periplasmic protein | 0.0785 | 0.0958 | -2.05 | NT |
| *Cj0633* | | putative periplasmic protein | 0.0000 | 0.0004 | -2.06 | NT |
| *Cj1593* | *rpsK* | 30S ribosomal protein S11 | 0.0000 | 0.0008 | -2.06 | NT |
| *Cj0671* | *dcuB* | anaerobic C4-dicarboxylate transporter | 0.0019 | 0.0093 | -2.13 | NT |
| *Cj0911* | | putative periplasmic protein | 0.0075 | 0.0208 | -2.17 | NT |
| *Cj1357c* | *nrfA* | putative periplasmic cytochrome C | 0.0004 | 0.0037 | -2.18 | NT |
| *Cj0021c* | | putative fumarylacetoacetate (FAA) hydrolase | 0.0005 | 0.0046 | -2.24 | NT |
| *Cj0854c* | | putative periplasmic protein | 0.0005 | 0.0046 | -2.28 | NT |
| *Cj0073c* | | conserved hypothetical protein Cj0073c | 0.0005 | 0.0043 | -2.30 | NT |
| *Cj0074c* | | putative iron-sulfur protein | 0.0000 | 0.0009 | -2.31 | NT |
| *Cj0909* | | putative periplasmic protein | 0.0008 | 0.0061 | -2.33 | NT |

| | | | | | | |
|---|---|---|---|---|---|---|
| *Cj0449c* | | conserved hypothetical protein | 0.0001 | 0.0018 | -2.33 | NT |
| *Cj0153c* | | putative rRNA methylase | 0.0009 | 0.0063 | -2.39 | NT |
| *Cj0473* | *nusG* | transcription antitermination protein | 0.0001 | 0.0018 | -2.40 | NT |
| *Cj1592* | *rpsM* | 30S ribosomal protein S13 | 0.0108 | 0.0265 | -2.48 | NT |
| *Cj0917c* | *cstA* | putative integral membrane protein | 0.0000 | 0.0012 | -2.55 | -5.5 |
| *Cj0910* | | putative periplasmic protein | 0.0011 | 0.0070 | -2.58 | NT |
| *Cj1358c* | *nrfH* | putative periplasmic cytochrome C | 0.0000 | 0.0004 | -2.73 | -6.7 |
| *Cj0893c* | *rpsA* | 30S ribosomal protein S1 | 0.0000 | 0.0004 | -2.74 | NT |
| *Cj0471* | *rpmG* | 50S ribosomal protein L33 | 0.0001 | 0.0014 | -2.87 | NT |
| *Cj0358* | | putative cytochrome C551 peroxidase | 0.0022 | 0.0102 | -2.89 | NT |
| *Cj0087* | *aspA* | aspartate ammonia-lyase | 0.0002 | 0.0023 | -3.05 | -5.8 |
| *Cj0864* | | putative periplasmic protein | 0.0003 | 0.0033 | -3.14 | NT |

NT, not tested
